# Defective DNA repair increases susceptibility to senescence through extension of Chk1-mediated G2 checkpoint activation

**DOI:** 10.1038/srep31194

**Published:** 2016-08-10

**Authors:** Yoshikazu Johmura, Emiri Yamashita, Midori Shimada, Keiko Nakanishi, Makoto Nakanishi

**Affiliations:** 1 Department of Cell Biology, Graduate School of Medical Sciences, Nagoya City University, 1 Kawasumi, Mizuho-cho, Mizuho-ku, Nagoya 467-8601, Japan; 2 Department of Perinatology, Institute for Developmental Research, Aichi Human Service Center, 713-8 Kamiya-cho, Kasugai, Aichi 480-0392, Japan; 3 Division of Cancer Cell Biology, Department of Cancer Biology, Institute of Medical Science, The University of Tokyo, 4-6-1, Shirokanedai, Minato-ku, Tokyo 108-8639, Japan

## Abstract

Susceptibility to senescence caused by defective DNA repair is a major hallmark of progeroid syndrome patients, but molecular mechanisms of how defective DNA repair predisposes to senescence are largely unknown. We demonstrate here that suppression of DNA repair pathways extends the duration of Chk1-dependent G2 checkpoint activation and sensitizes cells to senescence through enhancement of mitosis skipping. Extension of G2 checkpoint activation by introduction of the TopBP1 activation domain and the nondegradable mutant of Claspin sensitizes cells to senescence. In contrast, a shortening of G2 checkpoint activation by expression of SIRT6 or depletion of OTUB2 reduces susceptibility to senescence. Fibroblasts from progeroid syndromes tested shows a correlation between an extension of G2 checkpoint activation and an increase in the susceptibility to senescence. These results suggest that extension of G2 checkpoint activation caused by defective DNA repair is critical for senescence predisposition in progeroid syndrome patients.

Progeroid syndromes are a group of disorders characterized by clinical aspects mimicking physiological aging at an early age^1^. In general, the known progeroid syndromes are caused by mutations in genes encoding proteins involved in DNA repair or nuclear lamins, both of which result in a defect in specific DNA repair systems^2^,^3^,^4^,^5^. It is well documented that defects in DNA repair likely lead to age-related disorders^6^. Therefore, explorations of progeroid syndromes have shed light on DNA repair deficiency as a causal mechanism^7^. However, a molecular link between DNA repair deficiency and premature aging symptoms is still missing. The accelerated emergence of features of senescence is also a common characteristic in the known progeroid syndromes^8^. Senescence is believed to play pivotal roles in aging related changes as well as in suppression of carcinogenesis *in vivo*^9^,^10^,^11^. However, molecular mechanisms underlying senescence predisposition in progeroid syndrome patients remains elusive.

Activation of DNA damage response pathways is critical for induction of senescence^12^. Cellular responses to DNA damage are coordinated primarily by two signaling cascades, namely the ATM-p53-p21 and the ATR-Chk1-Cdc25 axes^13^, the former of which is known to be essential for senescence induction^14^ and the latter induces cell cycle arrest at G2 phase^15^. Importantly, the induction of p21 by activation of the former axis takes much longer time (at least several hours) than Cdk1 inhibition by the latter pathway (within 30 min), meaning that these two axes function at different time frames. We have recently unraveled the molecular mechanism underlying senescence induction in which activation of p53 at G2 results in degradation and transcriptional repression of mitotic regulators by premature activation of APC/C^Cdh1^ and activation of pRb family proteins, respectively, leading to a mitosis skip and generation of tetraploid G1 cells^16^. Thus, these results suggest that factors regulating G2 progression play a crucial role in senescence induction upon p53 activation.In this study, we found that defective DNA repair extended the duration of Chk1-dependent G2 checkpoint activation and increased susceptibility to senescence through accumulating cells at G2 phase when p21 was induced. Through manipulating the duration of G2 checkpoint activation, we concluded that the duration of Chk1 activation directs the senescence switch. This notion is supported by the observation showing a correlation between extension of G2 checkpoint activation and an increase in susceptibility to senescence in fibroblasts from progeroid syndrome patients.

## Results

### Extension of G2 checkpoint activation and increase in the susceptibility to senescence by defective DNA repair

**Figure 1:**
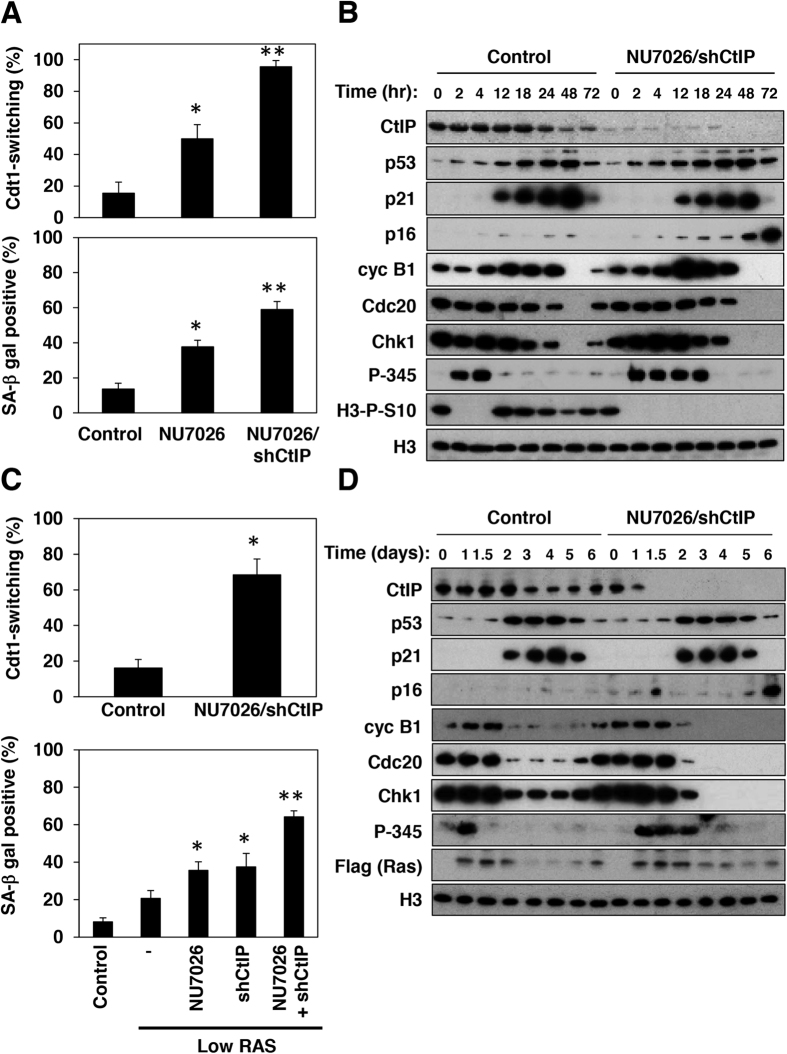
Defective DNA repair sensitizes cells to senescence upon IR irradiation and oncogene activation through extension of G2 checkpoint activation. (**A**) FUCCI-HCA2 cells expressing tet-on sh-control (Control) or tet-on sh-CtIP (shCtIP) were treated with doxycycline (1 μg/ml) for 24 hours. The treated cells were analyzed by time-lapse microscopy after IR treatment (2 Gy) in the presence or absence of 10 μM NU7026 for 3 days. The relative ratios of Cdt1-switching cells were determined by counting at least 100 cells. Data are presented as means ± s.d. of at least three independent experiments (upper panel). SA-β-gal-positive cells were identified at 6 days after treatment. Data are presented as means ± s.d. of at least three independent experiments (lower panel). *p < 0.01 vs. control (upper panel), *p < 0.001 vs. control (lower panel), **p < 0.001 vs. control and vs. NU7026. (**B**) Lysates from cells treated with either sh-control (Control) or sh-CtIP and NU7026 at the indicated times after IR treatment were subjected to immunoblotting using the indicated antibodies. (**C**) FUCCI-HCA2 cells expressing a high or low level of Ras^Val12^ with tet-on sh-control (Control) or tet-on sh-shCtIP (shCtIP) were treated with doxycycline (1 μg/ml) for 24 hours. The resulting cells were then analyzed as in (**A**). The relative ratios of Cdt1-switching cells (upper panel) and SA-β-gal-positive cells at 8 days after treatment (lower panel) were determined as in (**A**). Data are presented as means ± s.d. of at least three independent experiments. *p < 0.001 vs. control (upper panel), *p < 0.01 vs. control (lower panel), **p < 0.001 vs. control and vs. NU7026 and shCtIP. (**D**) Lysates from cells treated with sh-control (Control) or sh-CtIP and NU7026 at the indicated times after addition of doxycycline were subjected to immunoblotting using the indicated antibodies.

Normal human diploid fibroblasts, HCA2, were treated with different doses of IR and using the Fucci system^17^, senescence induction and Cdt1-switching {cells showing geminin-positive cells (green color) turn Cdt1-positive (red color) without entry into mitosis}, were evaluated by SA-β-gal staining as well as expression of p16 as a marker of senescence and time-lapse imaging, respectively. Senescence of HCA2 cells was effectively induced with 5 Gy irradiation or greater, but was not induced with 2 Gy or less (Supplementary Fig. 1A). p16 induction was evident at 72 hours after treatment in cells treated with 10 Gy irradiation, but not in those with 2 Gy irradiation (Supplementary Fig. 1B). Consistent with this, the majority of cells treated with 10 Gy underwent Cdt1-switching whereas only a few made the switch with 2 Gy (Supplementary Fig. 1C). The most notable difference between 2 and 10 Gy irradiation was in the duration of activation of Chk1 as evaluated by its phosphorylation at serine 345^18^ (Supplementary Fig. 1B). Levels of p21 induction appeared to be comparable between these two doses of irradiation. Low and high levels of Ras^V12^ expression produced similar dose-dependent effects of senescent induction and Cdt1-switching to those observed in cells treated with IR irradiation (Supplementary Fig. 1D). Again, the duration of Chk1 activation appeared to be much longer in cells expressing a high level of Ras^V12^ than in cells expressing a low level, although levels of p21 induction were comparable (Supplementary Fig. 1E). Again, p16 induction was only evident in cells expressing a high level of Ras^V12^. In these experiments, the levels of low and high expressions of Ras^V12^ were about 20-fold and 110-fold than the level of endogenous Ras expression, respectively (Supplementary Fig. 1F). Thus, these results suggest that the duration of Chk1-dependent G2 checkpoint activation, and not the level of p21, directs the switch that determines whether cells undergo senescence.The efficiency of DNA repair likely affects the duration of Chk1-dependent G2 checkpoint activation. Suppression of non-homologous end-joining DNA repair (NHEJ) by treatment with a DNA-PK inhibitor^19^, NU7026, increased the population of cells that underwent Cdt1-switching (Fig. 1A, upper panel) and subsequent senescence (Fig. 1A, lower panel) after exposure to 2 Gy irradiation. Suppression of NHEJ extended Chk1-dependent G2 checkpoint activation (Supplementary Fig. 2). Concomitant inhibition of homologous recombination DNA repair (HR) by depletion of CtIP^20^,^21^ with NHEJ using NU7026 further increased Cdt1-switching and senescence on 2 Gy irradiation (Fig. 1A). Importantly, these forms of suppression resulted in an extension of Chk1-dependent G2 checkpoint activation, but did not affect p21 induction (Fig. 1B). The enhancement of senescence induction was further supported by an increase in the expression of p16 and a loss of histone H3 serine 10 phosphorylation (H3-P-S10), a mitotic maker, as well as a loss of mitotic regulators in cells treated with NU7026 and shCtIP. Similar results were also observed in cells expressing a low level of oncogenic Ras, with an increase in populations of cells showing Cdt1-switching and positive for SA-β-gal staining under concomitant inhibition of NHEJ and HR (Fig. 1C). Again, this dual inhibition further extended Chk1-dependent G2 checkpoint activation but did not affect the level and kinetics of p21 induction. Taken together, defective DNA repair likely extends G2 checkpoint activation and consequently sensitizes cells to Cdt1-switching and senescence.

### Extension of G2 checkpoint activation increases susceptibility to senescence

**Figure 2:**
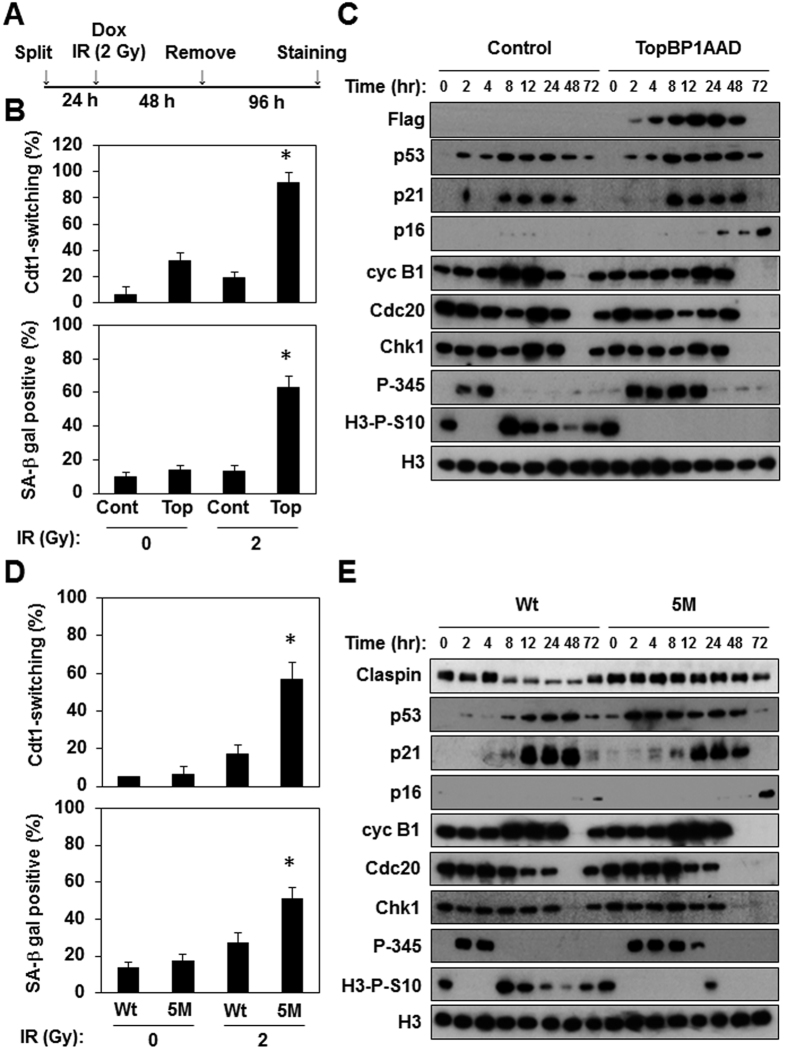
Extension of G2 checkpoint activation sensitizes cells to senescence upon exposure to a low IR dose. (**A**) Experimental outline of G2 checkpoint extension by transient expression of TopBP1AAD. Asynchronous FUCCI-HCA2 cells expressing tet-on 3xFLAG-TopBP1AAD were treated with IR (2 Gy) in the presence of doxycycline (1 μg/ml) for 48 hours, and then released into fresh medium and analyzed. (**B**) The relative ratios of Cdt1-switching cells (upper panel) and SA-β-gal-positive cells (lower panel) at 6 days after IR treatment were determined as in Fig. 1A. Data are presented as means ± s.d. of at least three independent experiments. *p < 0.01 vs. control (0 Gy), **p < 0.001 vs. control (2 Gy). (**C**) Lysates from cells treated with or without doxycycline at the indicated times after IR treatment were subjected to immunoblotting using the indicated antibodies. (**D**) FUCCI-HCA2 cells constitutively expressing wild type (Wt) or S30A/S34A/E90A/N91A/L92A mutant Claspin (5 M) were analyzed and the relative ratios of Cdt1-switching cells and SA-β-gal-positive cells at 6 days after IR treatment were determined as in (**A**). Data are presented as means ± s.d. of at least three independent experiments. *p < 0.001 vs. control (2 Gy). (**E**) Lysates of cells expressing wild type (Wt) or mutant (5 M) Claspin at the indicated times after IR treatment were subjected to immunoblotting using the indicated antibodies.

We then attempted to directly control the duration of Chk1-dependent G2 checkpoint activation through expression of either a TopBP1 ATR activation domain (AAD)^22^ or a nondegradable Claspin mutant^23^, the former being sufficient for ATR activation and the latter for suppression of recovery of the G2 checkpoint. We transiently expressed TopBP1 AAD for 48 hours in Fucci-HCA2 cells with a low level of DNA damage (Fig. 2A). This transient expression increased populations of cells showing Cdt1-switching and positive for SA-β-gal staining (Fig. 2B) specifically when the level of DNA damage was low. As expected, this expression extended the duration of G2 checkpoint activation, but did not affect p21 induction (Fig. 2C). Enhancement of senescence induction was further confirmed by a loss of mitotic regulators and H3-P-S10, and an increase in the expression of p16. Similar results were also observed when the nondegradable mutant of Claspin was expressed in cells with a low level of DNA damage (Fig. 2D,E).

### Shortening of G2 checkpoint activation decreases susceptibility to senescence

**Figure 3:**
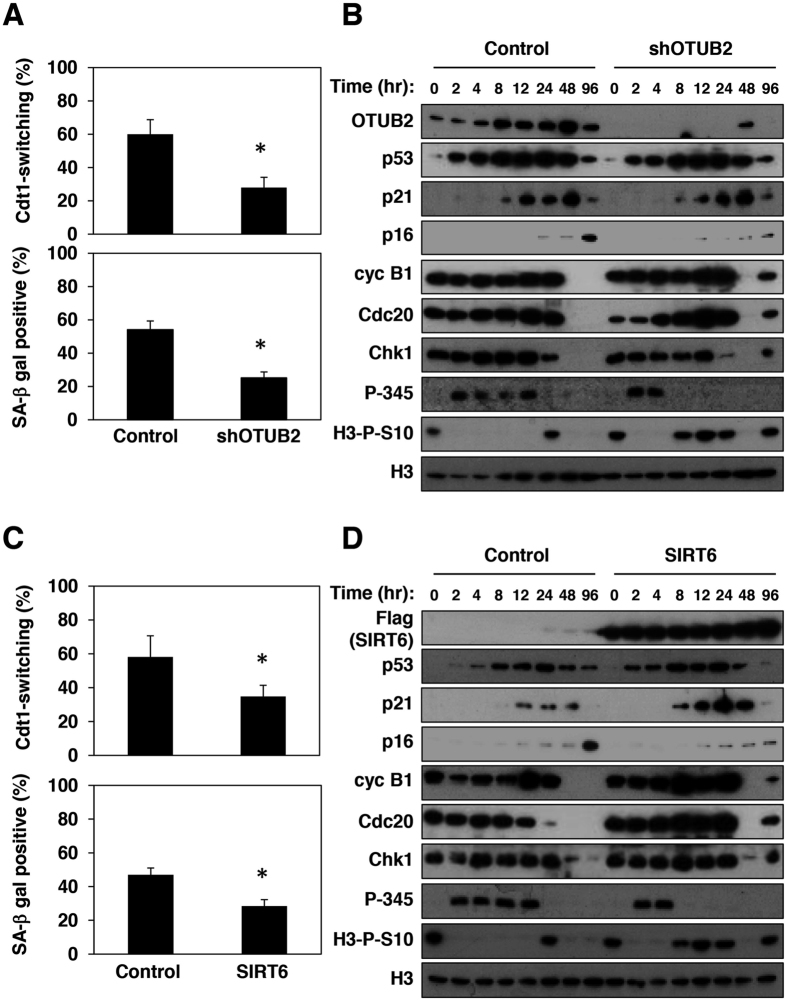
Shortening of G2/M checkpoint activation desensitizes cells to senescence upon exposure to a high IR dose. (**A**) FUCCI-HCA2 cells expressing tet-on sh-control (Control) or tet-on sh-OTUB2 (shOTUB2) were treated with doxycycline (1 μg/ml) for 24 hours, then analyzed 24 hours after IR treatment (5 Gy) and the relative ratios of Cdt1-switching cells and SA-β-gal-positive cells at 6 days after treatment were determined as in Fig. 1A. Data are presented as means ± s.d. of at least three independent experiments. *p < 0.01 vs. control. (**B**) Lysates from cells expressing sh-control (Control) or sh-OTUB2 (shOTUB2) at the indicated times after IR treatment were subjected to immunoblotting using the indicated antibodies. (**C**) FUCCI-HCA2 cells expressing tet-on 3xFLAG-Sirt6 (SIRT6) were treated with or without doxycycline (1 μg/ml) for 24 hours, then analyzed after IR treatment (5 Gy) and the relative ratios of Cdt1-switching cells and SA-β-gal-positive cells 6 days after treatment were determined as in (**A**). Data are presented as means ± s.d. of at least three independent experiments. *p < 0.01 vs. control. (**D**) Lysates from cells treated with or without doxycycline at the indicated times after IR treatment were subjected to immunoblotting using the indicated antibodies.

OTUB2 was recently reported to suppress DNA damage responses through suppression of deubiquitylation of ubiquitylated proteins with RNF8^24^. In addition, SIRT6 was reported to stimulate DNA double-strand breaks (DSBs) repair by activating PARP1^25^. When OTUB2 was depleted from Fucci-HCA2 cells, populations of cells showing Cdt1-switching and positive for SA-β-gal staining were reduced when a high level of DNA damage was present (Fig. 3A). OTUB2 depletion resulted in the shortening of G2 checkpoint activation (Fig. 3B). Loss of p16 induction, expression of mitotic regulators, and the presence of H3-P-S10 further confirmed the suppression of senescence induction in OTUB2-depleted cells. Similarly, when SIRT6 was expressed, Cdt1-switching and senescence induction were suppressed (Fig. 3C), together with a loss of p16 induction, expression of mitotic regulators, and the presence of H3-P-S10 (Fig. 3D). SIRT6 expression also shortened G2 checkpoint activation (Fig. 3D). Taken together, these results indicate that the duration of Chk1-dependent G2 checkpoint activation is a critical determinant of whether or not cells undergo senescence; that is, a shortened activation suppresses the induction of senescence whereas an extended activation enhances it.

### Correlation between extension of G2 checkpoint activation and high susceptibility to senescence in fibroblasts from progeroid syndrome patients

Impairment of a specific DNA repair system(s) is a prominent hallmark of most progeroid syndromes^26^. We thus expected that cells from progeroid patients would undergo extended Chk1-dependent G2 checkpoint activation and thus would be sensitized to senescence. DNA damage induced by IR is repaired by NHEJ or HR repair systems, whereas that induced by UV is mainly addressed by nucleotide excision repair or base excision repair^27^. Therefore, we examined the effects of IR and UV irradiation on Cdt1-switching and SA-β-gal-positive cells as well as the duration of G2 checkpoint activation in fibroblasts containing Fucci indicators from patients with various progeroid syndromes. Senescence as well as Cdt1-switching of HCA was induced by 30 J/m^2^ UV irradiation, but not by 10 J/m^2^ (Supplementary Fig. 3A). G2 checkpoint activation at 30 J/m^2^ UV was extended when compared with that at 10 J/m^2^ (Supplementary Fig. 3B).

**Figure 4:**
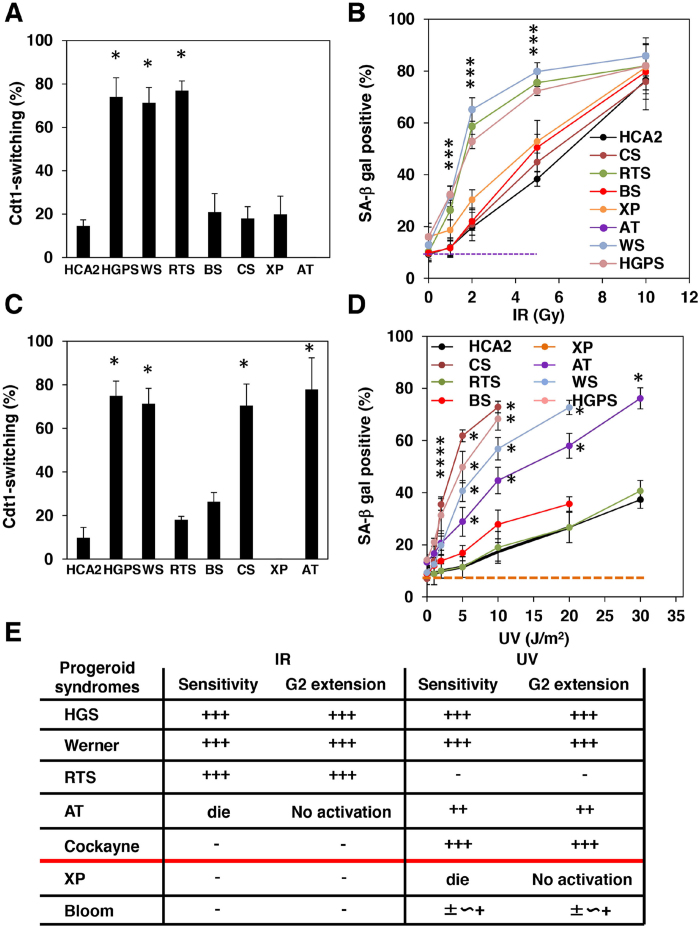
Fibroblasts from progeroid syndrome patients exhibit senescence sensitivity upon either or both IR or UV irradiation through extension of G2 checkpoint activation. FUCCI-HCA2 and FUCCI-fibroblasts from Hutchinson-Gilford Progeria Syndrome (HGPS), Werner Syndrome (WS), Rothmund-Thomson Syndrome (RTS), Bloom Syndrome (BS), Cockayne Syndrome (CS), Xeroderma pigmentosum (XP), and Ataxia Telangiectasia (AT) patients were analyzed by time-lapse microscopy after IR (2 Gy) (**A**,**B**) or UV (10 J/mm^2^)(**C**,**D**) treatment. The relative ratios of Cdt1-switching cells (**A**,**C**) and SA-β-gal-positive cells (**B**,**D**) at 6 days after treatment at the indicated doses were determined as in Fig. 1A. Data are presented as means ± s.d. of at least three independent experiments. *p < 0.001 vs. HCA2 (**A**,**C**) *p < 0.01 vs. HCA2 at the same doses of IR or UV. (**E**) Summary of senescence sensitivities and G2 checkpoint extension upon IR and UV in fibroblasts from progeroid syndrome patients.

Upon 2 Gy IR irradiation, the majority of cells from patients with Hutchinson-Gilford (HGPS) syndrome, Werner syndrome (WS), and Rothmund Thomson syndrome (RTS) underwent Cdt1-switching, whereas the majority from patients with Bloom syndrome (BS), Cockayne syndrome (CS), and Xeroderma Pigmentosum (XP) as well as normal HCA2 did not (Fig. 4A). Cells from an Ataxia telangiectasia (AT) patient died immediately after entry into mitosis, presumably due to impairment of DSBs-induced DNA damage responses^28^ (Fig. 4B and Supplementary Fig. 4). Consistent with this, cells from HGPS, WS, and RTS patients were highly sensitive to senescence even at a low level of IR irradiation, whereas those from BS, CS, and XP patients were not (Fig. 4B). Importantly, cells from HGPS, WS, and RTS patients specifically exhibited extended G2 checkpoint activation even at a very low level of IR irradiation, but those from BS, CS, and XP patients appeared normal. Intriguingly, extension appeared to correlate well with senescence sensitivity.In contrast, the majority of cells from HGPS, WS, CS, and AT patients underwent Cdt1-switching at 10 J/m^2^ UV irradiation, but those from RTS and BS patients as well as HCA2 cells did not (Fig. 4C). At this dose, cells from a XP patient died immediately after entry into mitosis due to a loss of Chk1-dependent G2 checkpoint activation (Fig. 4D and Supplementary Fig. 5). Consistent with this, cells from HGPS, WS, CS, and AT patients showed high sensitivity to senescence even at a low level of UV irradiation, whereas those from RTS and BS patients appeared to be comparable to HCA2 cells, although the BS cells were slightly sensitive (Fig. 4D). Again, cells from HGPS, WS, CS, and AT patients specifically exhibited extended G2 checkpoint activation and the extension appeared to correlate well with senescence sensitivity (Supplementary Fig. 5). In this respect, although BLM, the responsible gene for BS, is reported to have similar functions to WRN and RECQL4, the responsible genes for WS and RTS, respectively, cells from a BS patient showed a normal duration of G2 checkpoint activation on exposure to both IR and UV. Actually, BS is not a typical premature aging syndrome^29^. Taken together, the results suggest that extension of G2 checkpoint activation likely plays an important role in premature aging in progeroid patients. This idea was further supported by the observation that overexpression of SIRT6 in cells from a HGPS patient rescued the extension of G2 checkpoint activation and the senescence induction by IR irradiation (Supplementary Fig. 6A,B).

## Discussion

Our present results indicate that the duration of Chk1-dependent G2 checkpoint activation directs the molecular switch for senescence induction. Given that p21-induced premature activation of APC/C^Cdh1^ at G2 phase is necessary for a mitosis skip and senescence induction, and that transcriptional induction of p21 protein by p53 normally takes much longer than Chk1 activation in HDFs after treatment with senescence-inducing stimuli, our observations suggest that extended G2 checkpoint activation causes an accumulation of cells at G2 phase before induction of p21, leading to enhancement of a mitosis skip. Thus, all factors that affect the duration of Chk1-dependent G2 checkpoint activation, such as DNA repair and checkpoint recovery, likely regulate senescence induction.

Since G2 checkpoint activation and oncogene induced senescence are frequently found in precancerous lesions, but not in advanced cancer tissues, cellular senescence likely functions as an anti-tumorigenesis barrier *in vivo*^30^. Several lines of evidence demonstrate that proteins facilitating G2 checkpoint recovery are involved in human carcinogenesis^31^. For example, Polo-like kinase 1 (Plk1) whose activity is necessary for mitotic progression after G2 checkpoint recovery is overexpressed in various human caner tissues^32^. Wip1 and FoxM1 which play an important role in G2 checkpoint recovery are also overexpressed in human cancers^33^,^34^. Thus, the results suggest that a shortened Chk1-dependent G2 checkpoint activation could be involved in carcinogenesis *in vivo* through suppressing senescence induction. Intriguingly, cells from a XP patient with defects in their nucleotide excision repair system similar to those of CS, failed to undergo activation of the Chk1-dependent G2 checkpoint upon UV exposure^35^. Cells from an AT patient failed to activate the Chk1-dependent G2 checkpoint upon IR. Since XP and AT exhibit a predisposition to cancer, the results further support our idea that senescence suppression could play a key role in carcinogenesis *in vivo*.

Although the molecular basis for how defective DNA repair in progeroid syndrome patients accelerates the aging process has long been unknown, our observations offer important clues, particularly the correlation found between premature aging, extension of the G2 checkpoint, and senescence sensitivity upon a specific type of DNA damage (Fig. 4E). In this regards, recent reports suggest that accumulation of p16 positive cells in body accelerated age-related changes^36^ and limits healthy lifespan^37^. Thus, an extension of G2 checkpoint activation by impaired DNA repair likely accelerates senescence accumulation *in vivo*, leading to premature aging.

## Materials and Methods

### Plasmids

To generate lentivirus-based shRNA constructs, a 19–21 base shRNA-coding fragment with a 5′-ACGTGTGCTGTCCGT-3′ loop was introduced into pENTR4-H1 (a gift of Hiroyuki Miyoshi, RIKEN) digested with *Age*I/*Eco*RI. To insert the H1tetOx1-shRNA into the lentivirus vector, we mixed the resulting pENTR4-H1-shRNA vector and CS-RfA-ETBsd vector (a gift of Hiroyuki Miyoshi, RIKEN) with Gateway LR clonase (Invitrogen). All the target sequences for lentivirus-based sh-RNAs are summarized in Table S1.

To construct the Tet-on inducible lentivirus constructs, the *Bam*HI/*Not*I fragment of the PCR product containing cDNA for human SIRT6, NLS-TopBP1-AAD^38^, or H-RAS^Val12^ 39 was inserted into a pENTR-1A vector (Invitrogen) containing 3xFLAG epitope digested with *BamH*I/*Not*I. The resultant plasmid was mixed with CS-IV-TRE-RfA-UbC-Puro vector, and reacted with Gateway LR clonase to generate the lentivirus plasmid. The *Age*I/*Not*I fragment of the PCR product containing cDNA for human Claspin-WT or 5 M (S30A, S34A, E86A, E87A, N88A) fused to 3xFLAG epitope at the C-terminal region, was inserted into a CSII-CMV-MCS-IRES2-Bsd vector (a gift of Hiroyuki Miyoshi, RIKEN) digested with *Age*I/*Not*I.

### Immunoblotting analyses

Cells were directly lysed with Laemmli-buffer (2% SDS, 10% glycerol, 5% 2-mercaptoethanol, 0.002% bromphenol blue, and 62.5 mM Tris HCl at pH 6.8). The Cell lysates (20~50 μg) were separated by sodium dodecyl sulfate-polyacrylamide gel electrophoresis (SDS-PAGE), transferred to a Polyvinylidene Difluoride (PVDF) (Immobilon-P; Millipore) membrane, and then detected by immunoblotting with the indicated antibodies using enhanced chemiluminescence (ECL) detection. All antibodies used in this study are listed in Table S2.

### Virus generation and infection

Lentiviruses expressing the respective shRNAs or genes were generated by co-transfection of 293T cells with pCMV-VSV-G-RSV-RevB, pCAG-HIVgp, and the respective CS-RfA-ETBsd, CS-IV-TRE-RfA-UbC-Puro, or CSII-CMV-MCA-IRES2-Bsd using calcium phosphate co-precipitation. Cells infected with the indicated viruses were treated with 10 μg/ml of blasticidin (Invitrogen), 200 ng/ml of hygromycin (Sigma-puromycin (Sigma-Aldrich) for 2–3 days. Doxycycline (Sigma-Aldrich) was added to the medium at a concentration of 1 μg/ml for inducible expression of the respective shRNAs or genes.

### Cell culture

Early passage normal skin HDFs, HCA2^40^, and HDFs from patients with Rothmund-Thomson Syndrome (RTS-2, KURB1979, JCRB), Werner Syndrome (AG12795, Coriell Cell Repositories), and Hutchinson-Gilford Progeria Syndrome (AG06917, Coriell Cell Repositories), as well as HEK-293T cells (ATCC) were cultured in DMEM supplemented with 10% fetal bovine serum (FBS). HDFs from patients with Cockayne Syndrome (CS2AW, JCRB0310, JCRB), Bloom Syndrome (BS2CH, JCRB0317, JCRB), Xeroderma Pigmentosum (A) (XP35OS, JCRB0304, JCRB), and Ataxia Telangiectasia (AT2KY, JCRB0316, JCRB) were cultured with alpha-MEM supplemented with 10% FBS. All cells were cultured at 37 °C under 5% CO_2_, and treated with either IR or UVC at the indicated doses. Senescent cells treated with IR or UVC were analyzed 6 days after treatment and were evaluated by SA-β-gal staining. For oncogene-induced senescence, cells were infected with lentiviruses expressing tet-on 3xFLAG-H-Ras^Val12^ at an MOI of 1 (low RAS) or an MOI of 6 (high RAS), and were cultured in the presence of doxycycline at a concentration of 1 μg. The oncogene-induced senescent cells were analyzed 8 days after treatment and were evaluated by SA-β-gal staining as previously described^41^.

### Time-lapse microscopy

Cells expressing Fucci 2.1 indicators (pMXs-AmCyan-hGeminin (1/110) and pMXs-IP-mCherry-hCdt1 (30/120)) (a gift of Atsushi Miyawaki and Asako Sakaue-Sawano, RIKEN) were cultured on a glass bottomed dish (Iwaki) and placed on the stage of a BZ-9000 fluorescence microscope (Keyence) equipped with an environmental chamber (Keyence), which provided appropriate temperature, humidity, and CO_2_ conditions. Time-lapse images were captured every 20 minutes for 72 hours with a set of green 494/20 and 536/40 emission filters. Images were analyzed using BZ-9000 software. The relative ratio of Cdt1-switching cells versus the total number of cells changing from green to red was determined by counting at least 100 cells.

### Quantitative RT-PCR

Total RNA was extracted using ISOGEN II (Wako) according to the manufacturer’s instructions. For qRT-PCR analysis, cDNAs were synthesized using a SuperScript II cDNA synthesis kit (Invitrogen). Real-time PCR amplifications were performed in 96-well optical reaction plates with Power SYBR Green PCR Master Mix (Applied Biosystems). The relative expression values of each gene were determined by normalization to GAPDH expression for each sample. Primer sequences are available upon request.

### Statistical Tests

An unpaired *t* test was used when comparing two groups, whereas an analysis of variance (ANOVA) followed by Tukey-Kramer’s *post hoc* test was used when comparing three or more groups. All statistical analyses were performed using PC software (Instat 3, GraphPad Software, Inc., La Jolla, CA). Probability values less than 0.05 were regarded as significant.

## Additional Information

**How to cite this article**: Johmura, Y. *et al*. Defective DNA repair increases susceptibility to senescence through extension of Chk1-mediated G2 checkpoint activation. *Sci. Rep.* **6**, 31194; doi: 10.1038/srep31194 (2016).

## Supplementary Material

Supplementary Information
