# Supplementary material for: Defective DNA repair increases susceptibility to senescence through extension of Chk1-mediated G2 checkpoint activation
Source: Sci Rep. 2016 Aug 10;6:31194. doi: 10.1038/srep31194 (PMC4979019; doi:10.1038/srep31194)
Supplement: Supplementary Information [file srep31194-s1.pdf]

Defective DNA repair increases susceptibility to senescence through extension of Chk1-mediated G2 checkpoint activation

Yoshikazu Johmura, Emiri Yamashita, Midori Shimada, Keiko Nakanishi and Makoto Nakanishi*

* E-mail: mkt-naka@ims.u-tokyo.ac.jp

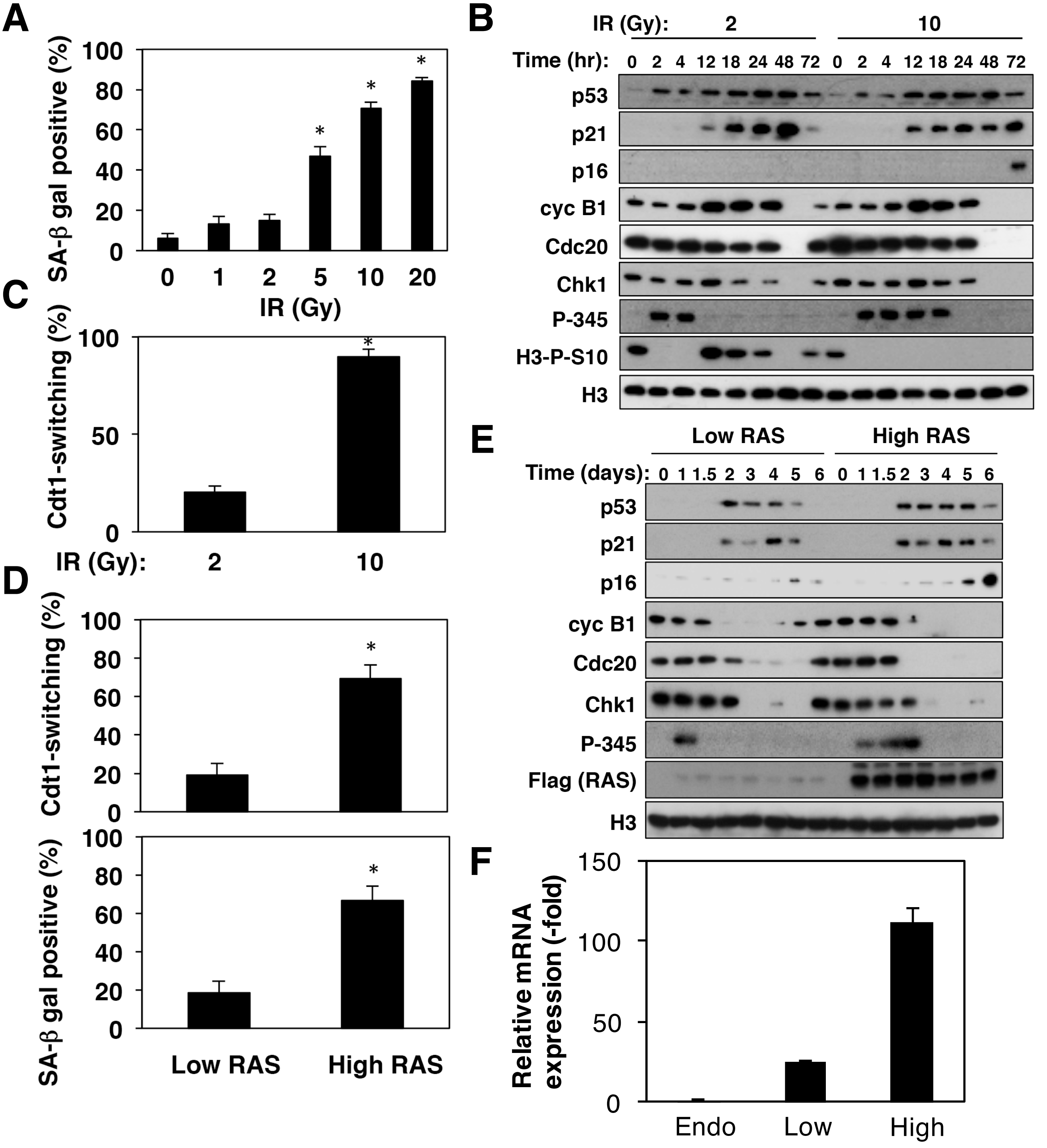

Supplementary Figure 1 Dose-dependent induction of IR- and oncogene-induced senescence (A) HCA2 cells were treated with the indicated doses of IR. SA-β-gal positive cells were identified at 6 days after treatment. Data are presented as means±s.d. of at least three independent experiments. **(B)** Lysates from HCA2 cells at the indicated times after IR (2 or 10 Gy) treatment were subjected to immunoblotting using the indicated antibodies. **(C)** FUCCI-HCA2 cells were treated with IR (2 or 10 Gy), then analyzed and the relative rations of Cdt1-switching cells were determined as in Fig. 1A. Data are presented as means±s.d. of at least three independent experiments. **(D)** FUCCI-HCA2 cells expressing tet-on high or low level of Ras^{V12} were treated with doxycyclin (1 μg/ml), then analyzed, and the relative ratios of Cdt1-switching cells (upper panel) and SA-β-gal-positive cells (lower panel) at 8 days after treatment were determined as in **(A)**. Data are presented as means±s.d. of at least three independent experiments. **(E)** Lysates of cells treated with doxycycline at the indicated times were subjected to immunoblotting using the indicated antibodies. **(F)** Cells as in **(D)** were treated with or without (Endo) doxycyclin (1 μg/ml), and then their total RNAs at 48 hrs were subjected to qPCR analysis using a primer set for endogenous Ras. *p<0.001 vs. 0 Gy **(A)**, 2 Gy **(C)**, and Low Ras **(D)**

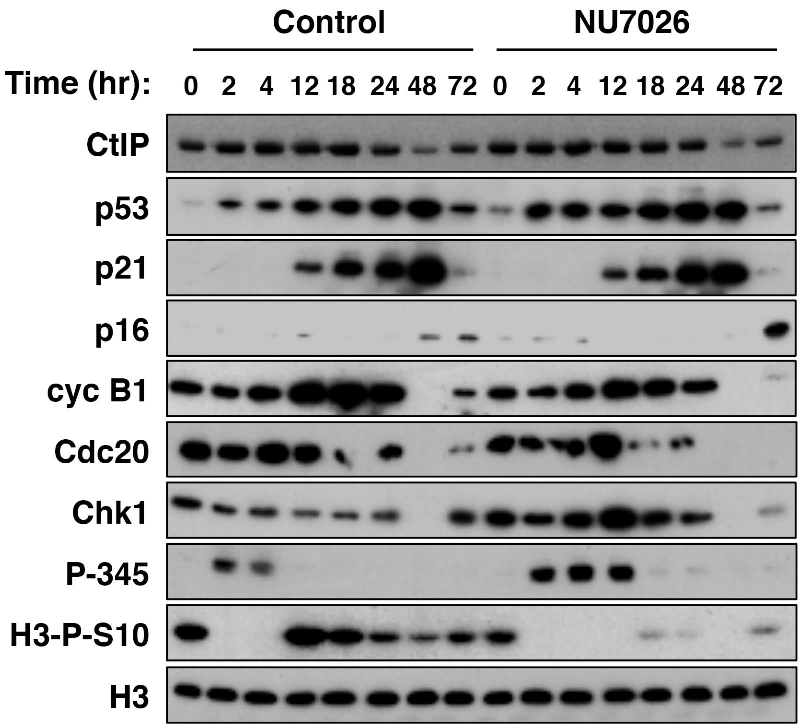

Supplementary Figure 2 Impaired NHEJ DNA repair partially sensitized cells to senescence through G2 checkpoint extension The lysates from HCA2 cells expressing sh-luciferase in the presence or absence (Control) of NU7026 at the indicated times after IR (2 Gy) treatment were subjected to immunoblotting using the indicated antibodies.

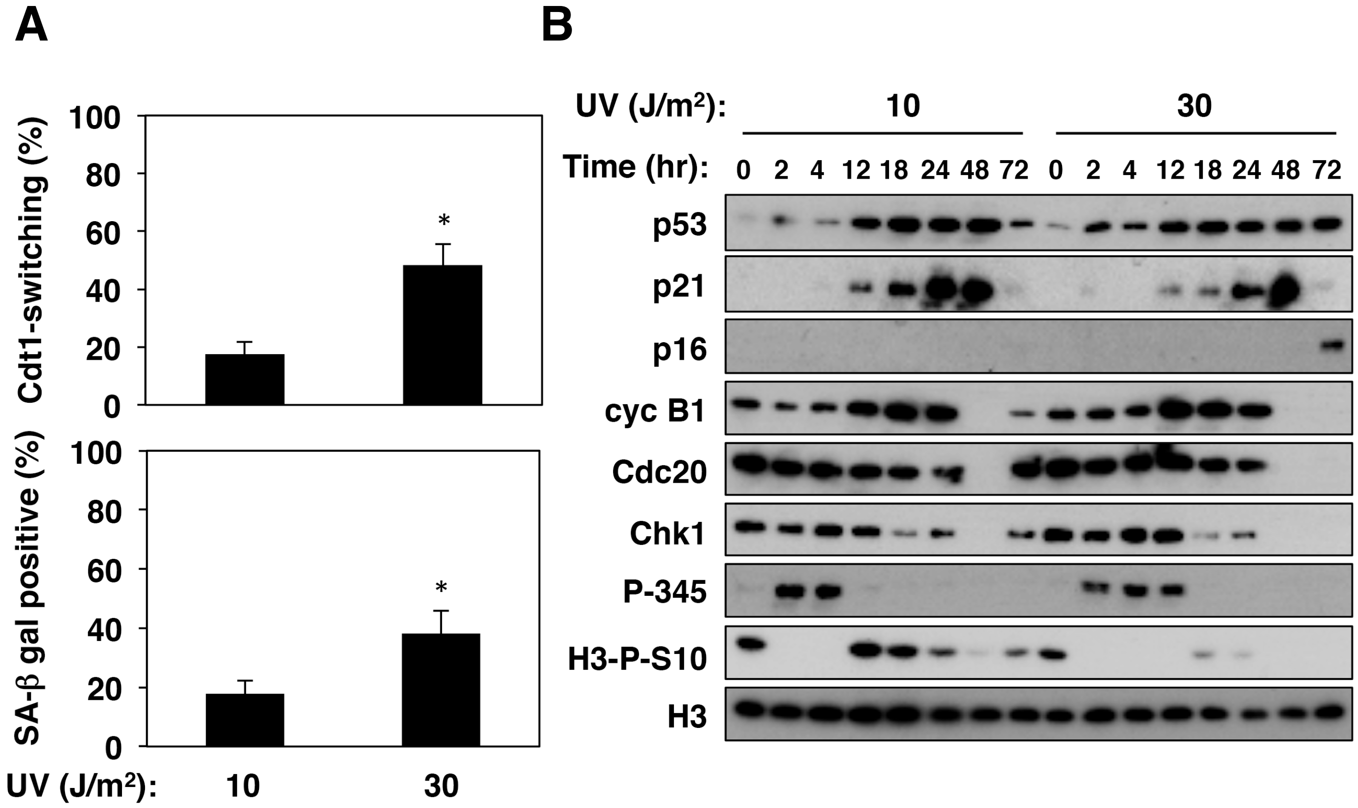

Supplementary Figure 3 Dose-dependent induction of senescence on UV irradiation **(A)** FUCCI-HCA2 cells were treated with UV (10 or 30 J/m^2), then analyzed , and the relative rations of Cdt1-switching cells (upper panel) and SA-β-gal-positive cells at 8 days after treatment were determined as in Fig. 1a. Data are presented as means±s.d. of at least three independent experiments. *p<0.01 vs. 10 J/m^2 **(B)** Lysates from cells treated with UV (10 or 30 J/m^2) at the indicated times were subjected to immunoblotting using the indicated antibodies.

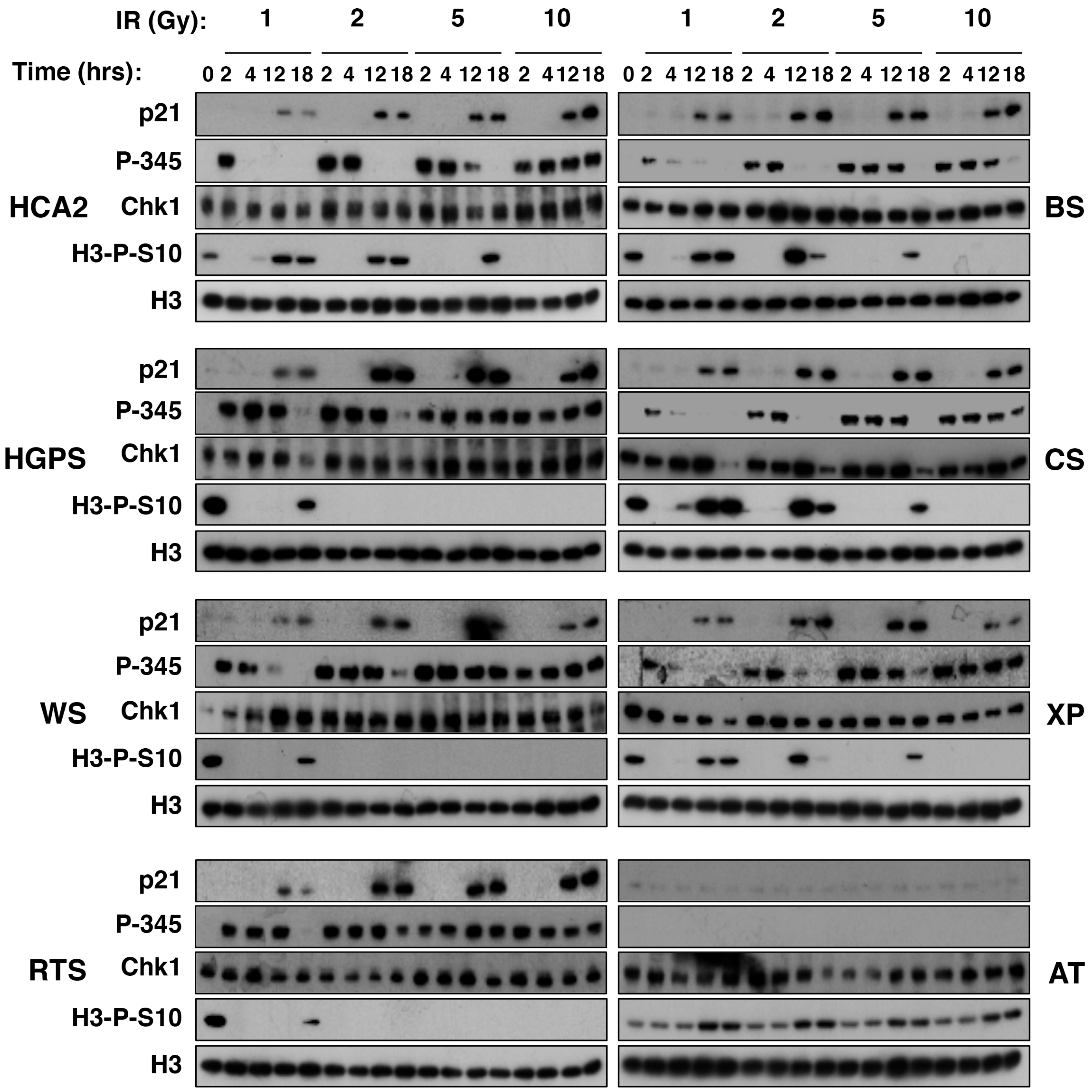

Supplementary Figure 4 Senescence sensitivity of fibroblasts from progeroid syndrome patients on IR treatment through extension of G2 checkpoint activation
Lysates of HCA2 or fibroblasts from progeroid syndrome patients collected at the indicated times after distinct doses of IR (0, 1, 2, 5, 10 Gy) were subjected to immunoblotting using the indicated antibodies.

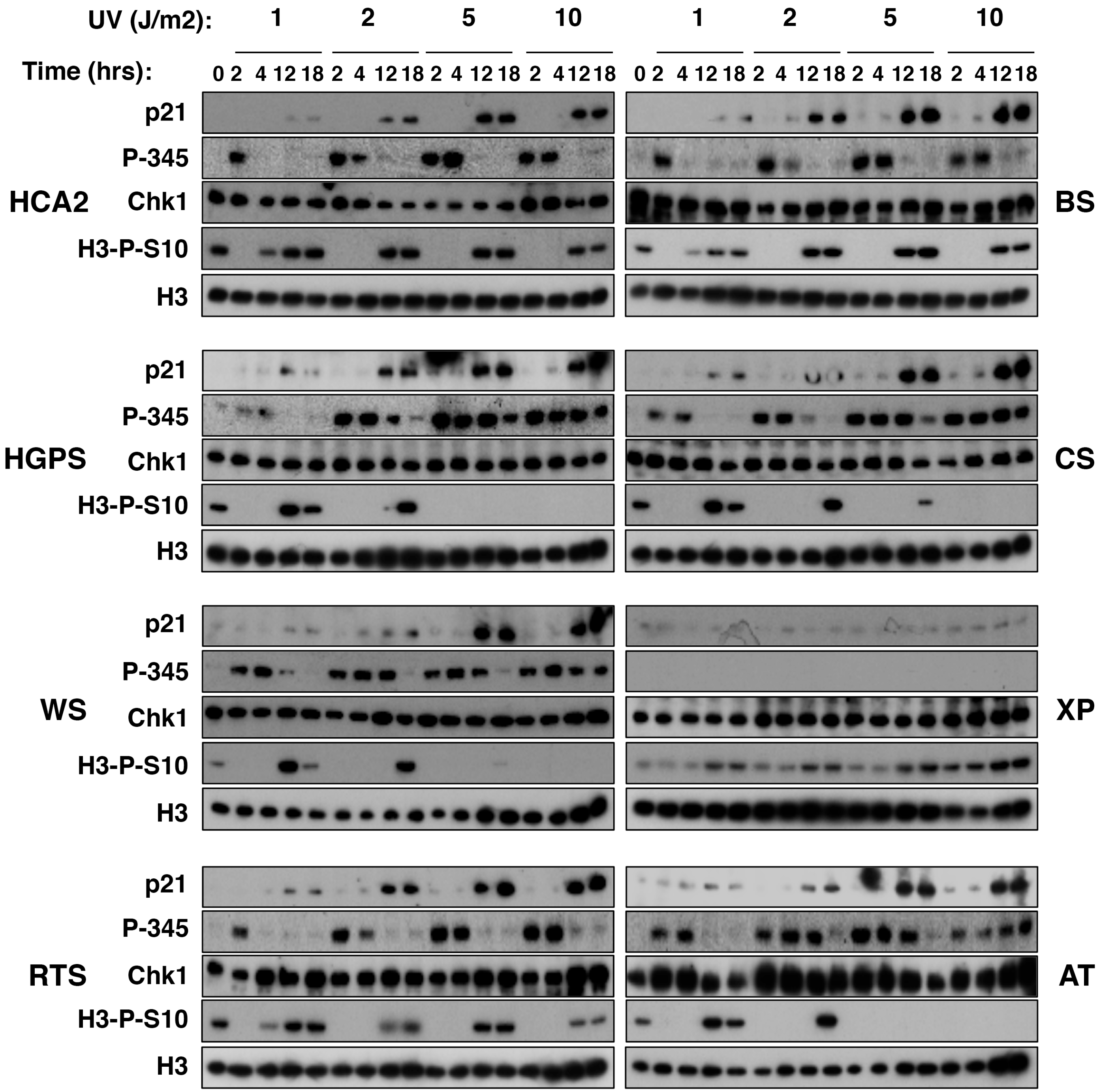

Supplementary Figure 5 Senescence sensitivity of fibroblasts from progeroid syndrome patients on UV treatment through extension of G2 checkpoint activation
Lysates of HCA2 or fibroblasts from progeroid syndrome patients collected at the indicated times after distinct doses of UV (0, 1, 2, 5, 10 J/m^2) were subjected to immunoblotting using the indicated antibodies.

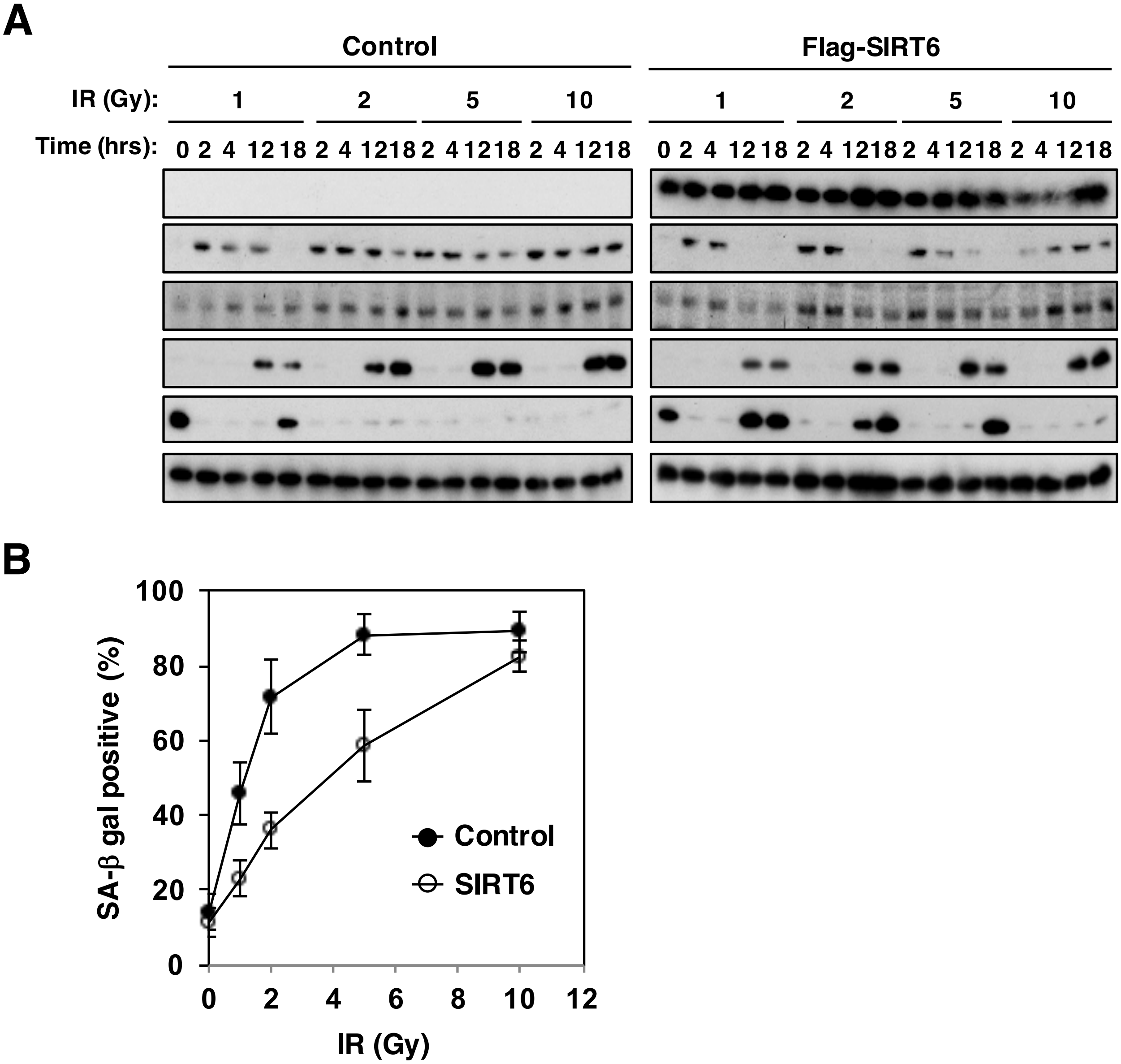

Supplementary Figure 6 SIRT6 expression partly rescues the extension of G2 checkpoint activation and senescence induction by IR irradiation in HGPS cells
(A) Fibroblasts from a HGPS patient expressing tet-on 3xFlag-SIRT6 were treated with or without doxycyclin (1 μg/ml) for 24 hrs, irradiated with IR at the indicated doses. Lysates at the indicated times were subjected to immunoblotting using the indicated antibodies. **(B)** Fibroblasts as in **(A)** were irradiated with IR at the indicated does and subjected to SA-β-gal staining 6 days after irradiation. SA-β-gal positive cells were determined as in Fig. 1A. Data are presented as means±s.d. of at least three independent experiments.

Table S1. shRNA sequences used in this study

Target gene	Sequence	Reference	Type
CtIP	GCATCATCCTTCAGCCCTTGA	(Hirokawa et al., 2014)	lentivirus
Luciferase	CGTACGCGGAATACTTCGA	(Elbashir et al., 2001)	lentivirus
OTUB2	CAGAGUGCCUCGGACCACA	(Kato et al., 2014)	lentivirus

Table S2. Antibodies used in this study

Antibodies	Species	Source
Anti-CDC20 (E-7)	Mouse	Santa Cruz Biotechnologies, Santa Cruz, CA
Anti-Chk1 (c9358)	Mouse	Sigma, St. Louis, MO
Anti-Chk1 P-345 (2348)	Rabbit	Cell Signaling Technology, Boston, MA
Anti-Claspin (H-300)	Rabbit	Santa Cruz Biotechnologies, Santa Cruz, CA
Anti-CtIP (D-4)	Mouse	Santa Cruz Biotechnologies, Santa Cruz, CA
Anti-Cyclin B1 (GNS1)	Mouse	Santa Cruz Biotechnologies, Santa Cruz, CA
Anti-FLAG (M2)	Mouse	Sigma, St. Louis, MO
Anti-Histone H3 (ab1791)	Rabbit	Abcam, Cambridge, United Kingdom
Anti-Histone H3 P-S10 (9701)	Rabbit	Cell Signaling Technology, Boston, MA
Anti-p16 (JC8)	Mouse	Santa Cruz Biotechnologies, Santa Cruz, CA
Anti-p21 (F-5)	Mouse	Santa Cruz Biotechnologies, Santa Cruz, CA
Anti-p53 (DO-1)	Mouse	Santa Cruz Biotechnologies, Santa Cruz, CA
